# A new posture-correcting system using a vector angle model for preventing forward head posture

**DOI:** 10.1080/13102818.2014.949040

**Published:** 2014-10-27

**Authors:** Hojun Yeom, Juhun Lim, Sung Hak Yoo, Woocheol Lee

**Affiliations:** ^a^ Department of Bio Medical Engineering, Eulji University, Seongnam-si, Gyeonggi-do, Korea

**Keywords:** forward head posture, vector model, functional electronic stimulation, digitized measurement

## Abstract

In modern society many people are afflicted with muscle pain in the neck and shoulders mainly caused by incorrect posture. The number of patients having neck pain is increasing as usage of digital devices becomes more frequent. If patients could be notified how inappropriate their postures are in real time, the number of patients could be lower. Unfortunately, there is no digitized standard way of diagnosis for forward head posture. This study applies a concept based on a vector related to two angles which are acquired from the neck and the head, so that a device can diagnose the posture by measuring and analysing the angles. To obtain the vector, integral calculations of displacement of the head are needed. As a result, with this device, patients’ faulty posture can be easily detected.

## Introduction

It is well known that in the pattern of modern life the use of digital devices has become very frequent. According to the Kaiser Family Foundation, 8- to 18-year olds generally spend about 8 hours using ‘entertainment media’ every day.[1] As Cisco VNI (Cisco® Visual Networking Index), which designs, manufactures and sells networking equipment, reported, the global number of digital devices will be over 2 billion by 2018.[2] At the same time, many articular diseases related to the upper part of the spinal column, especially forward head posture (FHP), are also increasing rapidly. Recently, a new term ‘text neck’ has even been introduced in connection with using mobile devices such as cell phones and laptops. The Chiropractors’ Association reports that dozens of cases of ‘text neck’, including FHP, are being reported each week.[3] They have investigated a number of patients with FHP in various age groups. A quarter of the patients in the group aged 5–10 were diagnosed with FHP. However, the number increased three times in the group aged 11–16.[4]

According to Kapandji,[5] for every 2.5 cm the head moves forward, it gains 0.45 kg in weight, as far as the muscles in the upper back and neck are concerned, because they experience more strain to support the position of the head. An alarming fact is that FHP may result, according to Calliet,[6] in the loss of up to 30% of vital lung capacity.

However, even though a lot of critical effects are known to be caused by incorrect posture and FHP, most people do not pay attention to their posture in their everyday life, which can aggravate their articular disease (usually of the neck or back). Since there is no digitized-standard for diagnosis of FHP, only a doctor can determine whether a patient's posture is correct. Due to the lack of digitized-standard and attention to FHP, we suggest a new system for diagnosis and correction of faulty posture is needed.

## Materials and methods

### Normal and correct posture

As the head is supported by the neck bones and muscles, the neck should sustain the whole weight of the head. From the perspective of structure, there is a proper position range for keeping the head effectively straightened. However, there is no such real static position for a moving head in response to every motion. Generally, a person's head weighs about 4.5–5.5 kg, which is rather heavy for sustaining for a long time.

**Figure 1.**
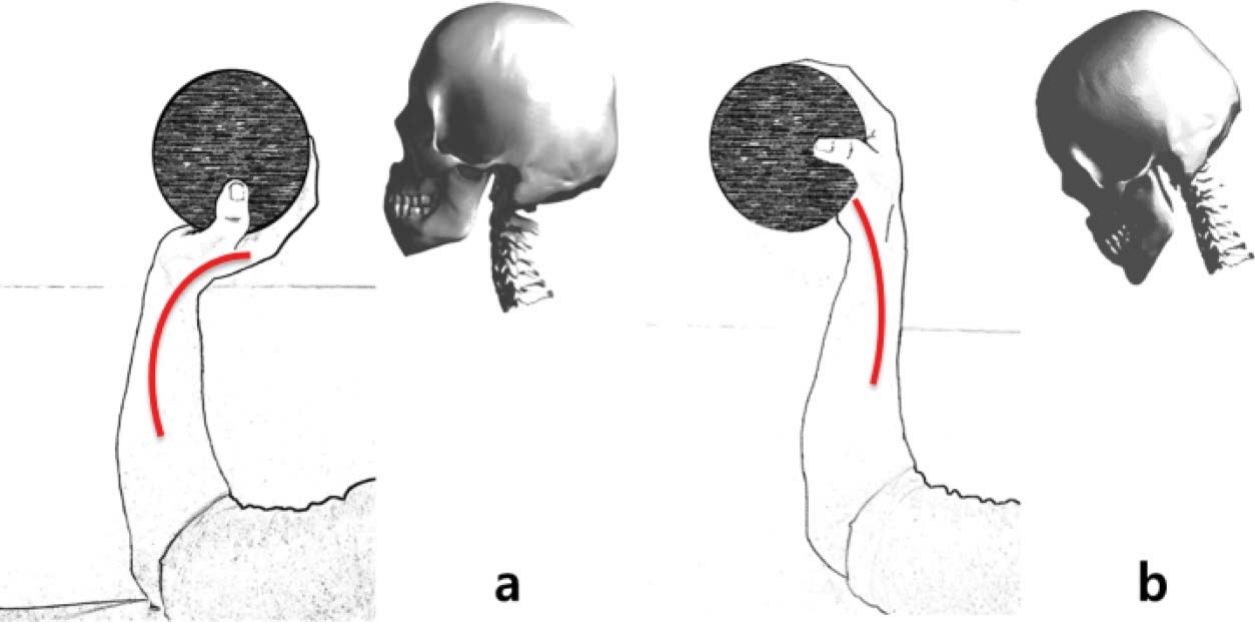
A model drawing a parallel between the head position and an arm holding up a ball. Correct position (a) and incorrect position (b).

The structure of the neck and the head can be demonstrated with an arm holding a ball weighing as much as a human head. While the hand is holding the ball, normally we can see a C-shape on the neck as shown in Figure 1(a). If the mass shifts forward and the angle of the neck changes, the configuration becomes much more stressful on the wrist as shown in Figure 1(b). The bones and muscles around the wrist have to hold the weight at the cost of higher tension and strength due to affect relating to moment.

**Figure 2.**
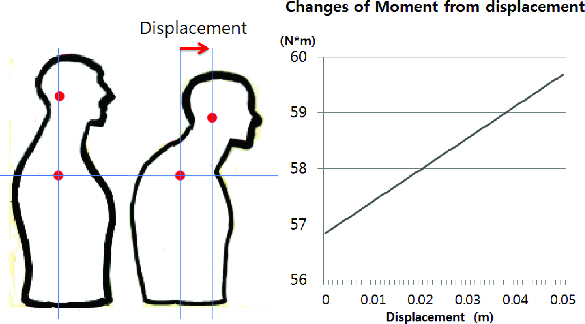
Changing displacement of head causes changes in moment.

Figure 2 shows how the weight of the head begins to increase as the head moves forward. Therefore, the positions of the head and the curve of the neck have to be mechanically in harmony to reduce any unnecessary stressful load that can cause neck pain.[7] A proper posture can be easily described with two spots, the centre of one ear and the edge of a shoulder. When the two points lie in a straight line that is at a right angle to the ground, the mass is properly supported; this is the normal posture as shown in Figure 2.

### Diagnosis of FHP in practice

#### Method 1

FHP is a condition in which the neck and the head rest in front of, rather than in line with, the shoulder. A simple way of testing the posture is by using a pair of sliding callipers to measure the displacement of the horizontal axis between the centre of the ear and the shoulder.

As illustrated in Figure 2, on the left side, it is considered a good posture when the line running through the ear to the middle of the shoulder is perpendicular to the ground. In the middle panel, however, the head has leaned forward and the ear lobe does not line up.[8] This type of displacement causes changing moment of the head. Figure 2 also demonstrates how the moment that the neck should sustain is increased by various displacements of the head. Table 1 shows the degree of FHP classified by measurement of the displacement of the head.

**Table 1.** Classified magnitude of FHP by displacement.

Displacement	Degree of FHP
1–2.5 cm	Slight FHP
2.5–5 cm	Severe FHP
5 cm	Highly severe FHP

#### Method 2

Advanced studies on FHP suggest an approach for diagnosis of FHP through measurement of the craniovertebral (CV) angle.[9,10] It is reported that the CV angle is closely related to one of the causes for neck pain, as poor postural awareness and habitually poor postures may result in greater loads on the sustaining structure and may cause sensitization and pain. A smaller CV angle indicates that the degree of FHP is severe.[11] Generally, in FHP patients the averaged CV angle measured is lower than 45°.[12]

### Correcting methods of FHP

#### Conventional method

Some cases of FHP are caused by stiffness in the muscles of the frontal neck. When the muscles contract, this causes a postural imbalance, pulling the head and moving the neck forward. The first way of correcting the posture is by stretching these tight muscles, which can help the head return more easily to a neutral position. There are some manual processes that are commonly used in practice. They are stretching, back and neck exercises, changing positions, muscle and postural re-education. These are also applied to healthy people, not only patients, manually by professionals. According to physicians, the most important process is the so-called ‘re-education’ for effective adjustment of the posture. In this step, with correctly educated posture, people should continue trying to maintain good posture for preventing FHP or stopping the neck pain from aggravating.[13,14]

Second, there is also a method of surgical correction. If a patient suffers from extreme pain the neck or arm, a surgery may be offered to decrease the pain. This procedure is usually done in order to make the neck stable by refilling the space between the vertebrae with a bone implant. If a patient's neck bones lean forward, there is no C-shape, rather the neck is straightened up. After an operation to refill the space with plates, the neck will regain its normal condition. Even though this surgical procedure poses a high degree of risk because the nerve might be damaged, the results are reasonably promising. Chemotherapy and radiation therapy studies are ongoing as well.[15,16]

#### Suggestion of a new method

There are many kinds of electric stimulators for reducing pain caused by incorrect posture. However, the results from the pain relief treatment that is currently used could be improved by way of digital diagnosis. In addition, the stimulators do not have an adjusting function to the fundamental problem. If a system is designed to give feedback to patients, it would give consequentially superior results.[17] That is why this study proposes a new system, functional electrical stimulation (FES), for correcting posture based on a mathematically digitized vector.

### A new method of diagnosis FHP

#### Concept of vector design

Regardless of the actual cause of neck pain, it should be expected to be rooted in the structure of the neck and muscles. A simple way of schematization of body structure is to set a vector.[18] The vector is the total sum of all measured axis vectors.[19] For drawing the vector of direction, there has to be a reference point. For the reference of a vector, the first cervical vertebrae can be effectively used, as many studies recommended.

An arrow will be aligned from this starting point (also called reference point) to a target point where the sensor indicates.

**Figure 3.**
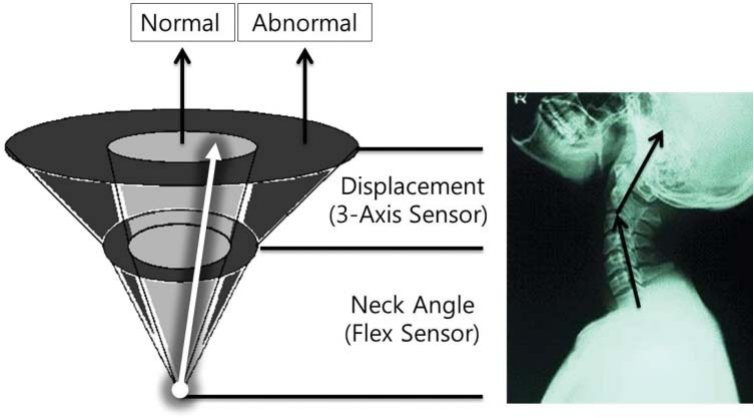
Vector-sum is composed of angles of structure.

Since the vector acquired from an acceleration sensor refers to the position of the head, comparing with the position of the first vertebrae, there is still a lack of data for the angle of the neck. By combining another sensor, Flex Sensor, the system can accurately measure the angle of the neck which might be influenced by any movements of the head. Furthermore, through a few calculations, the system can embody the vector in an imaginary three-dimensional structure. Because it is mathematically calculated, it can be considered as a digitized standard for diagnosis of FHP. Once the vector shifts from the normal range to an abnormal area (calculated based on a medical standard), this indicates that the posture is improper and can be considered as FHP (Figure 3).

After measurement and analysis, the device can cope with this abnormal posture by itself, which is the main purpose of this system. In fact, further research on setting vectors has to be done for more accurate diagnosis of FHP. Moreover, there are many variables in setting a vector. Nonetheless, this system suggests the combination of two methods which have been often used so far.

#### New approach using displacement and neck angle

The well-calibrated acceleration sensor provides information about the slope where it locates. In this study, a three-axis accelerator sensor is located at the right side of the ear. This slope data will be converted into displacement by double integration. As the sensor module moves, the displacement means how much far the head has moved/slid. By checking the displacement of the module, the device judges whether the posture is incorrect and how bad it is. The three-axis sensor detects accelerations in three directions, meaning that the device can even measure coronal head tilt.[20,21] (Table 2).

**Table 2.** Voltage (mV) corresponding to the sensors.

		Three-axis acceleration sensor		
Sensor degree (′)	Flexible sensor	*X*-axis	*Y*-axis	*Z*-axis
−60	1065	917	943	950
−50	1117	996	998	996
−40	1187	1119	1113	1113
−30	1273	1213	1216	1204
−20	1345	1316	1312	1335
−10	1443	1453	1461	1486
0	1500	1590	1622	1634
10	1655	1740	1768	1778
20	1720	1841	1911	1883
30	1811	1966	2034	1995
40	1903	2098	2141	2137
50	1998	2196	2261	2257
60	2112	2290	2320	2324

Not only is FHP important to be detected, but also many kinds of vertebral diseases can be diagnosed by the sensor. However, using only the three-axis accelerator module is not accurate enough because wearing a sensor on one side of the ear, given the human body structure, cannot detect the slope of the neck.

**Figure 4.**
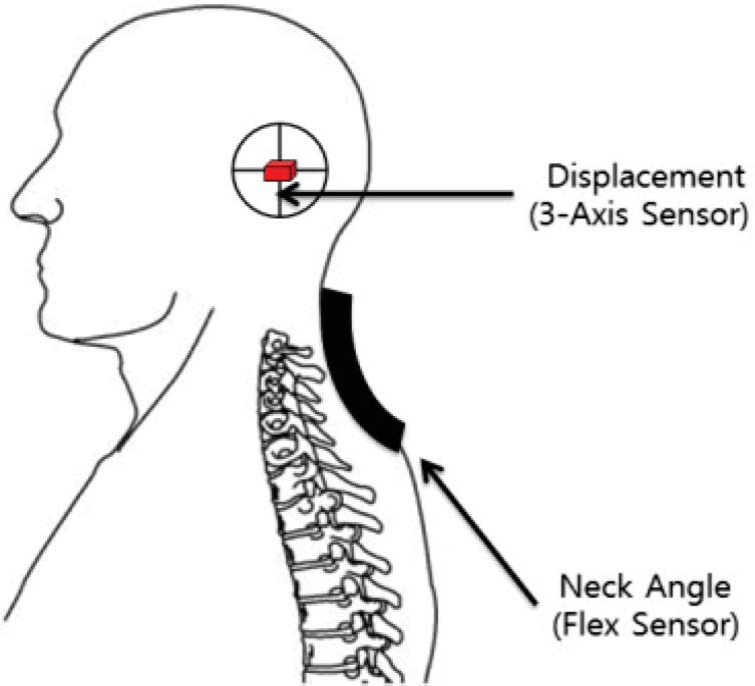
Locations of the two sensors.

A flex sensor positioned at the rear or the neck can be useful for providing additional references. Being able to detect the angle of the neck makes the system more sensitive for detecting backward and forward motion. The two sensors are placed as represented in the diagram (Figure 4), which is based on the fact that the CV angle is the index that shows how the neck is bent. The flex sensor module gives the microprocessor the data about the angle of the neck in the form of direct current (DC) voltage. Upon bending of the neck, both the angle and the voltage output change. Table 2 shows the voltage output corresponding to changes in the angle.

##### Three-axis acceleration sensor element

AM-3AXIS Ver.03, manufactured by NewTC, has 10 pins, including power source pins and data transfer pins. Specifically *X*-, *Y*- and *Z*-axis analogue data pins can be connected to a microprocessor so that the micro-processor operates some calculations. Analogue data from each axis indicate how the module moves and how it is tilted. This sensor module can be designed to be significantly smaller so it could be worn like an earphone. With a few mathematical operations and electronic circuits, the needed data, such as acceleration, velocity and displacement of module, can be obtained.

##### Strain gauge (FLEX SENSOR)

The flex-sensor patented technology is based on resistive carbon elements. As a variable printed resistor, the flex sensor achieves great form-factor on a thin flexible substrate. When the substrate is bent, the sensor produces a resistance output: the more bent it is, the higher the resistance value is (10–20 K). Two strain gages are used in a full-bridge circuit for linear operating.[22]

The combination of two different sensors enables the system to detect the circumstances sensitively, with whole steps being mathematically digitized so that the system can possibly diagnose FHP accurately. The changes of voltage from each axis are reasonably similar in shape, meaning that they are in a linear proportional relationship. Therefore, the data from each axis can be directly used for measuring the angle without additional steps.

**Figure 5.**
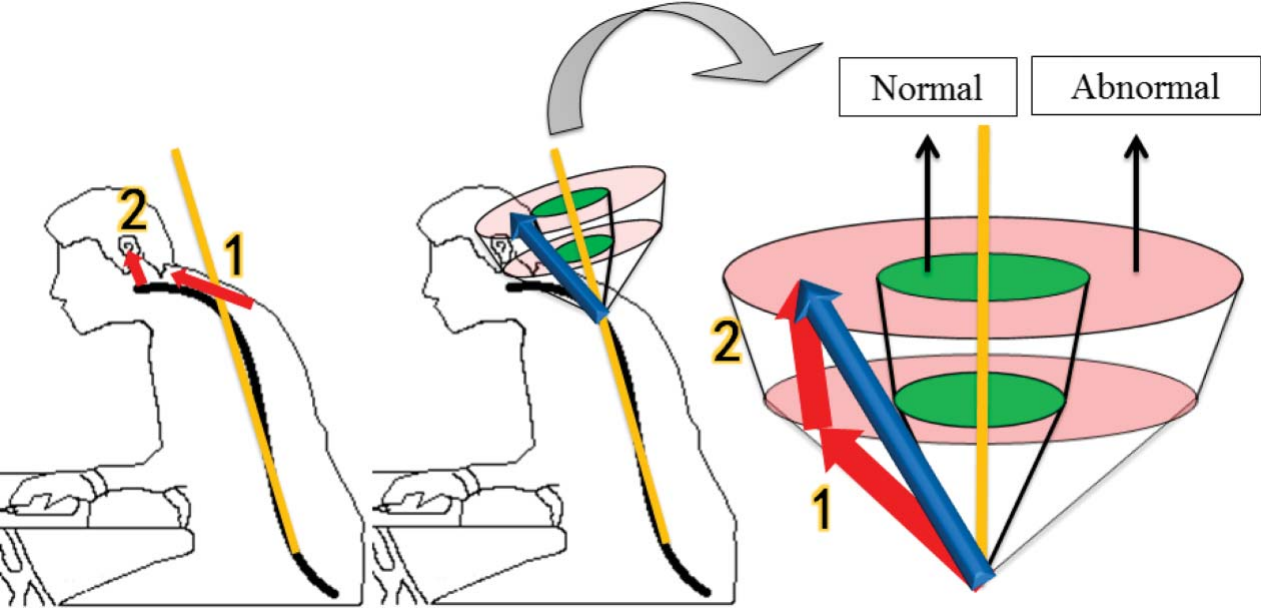
Vector model on human body.

Figure 5 shows a digitized-standard concept that determines if a measurement result falls within the normal range. The two angles in the diagram need to be defined and confirmed by further research with medical evidence. For the purposes of the present study, however, the total normal range was assumed to have a 36° angle by actual experiment. Its range is reasonably supported by an advanced study on the CV angle that shows that the CV angle is much smaller than 45° in most FHP patients.[13]

Angle 1 is measured by the flex sensor and angle 2 is calculated from information provided by the three-axis acceleration sensor (Figure 5). These two angles are converted to a vector model. The sum of the two angle vectors is generated and displayed in the form of a vector model. As a result, the total vector signals that the posture is substantially tilted in the abnormal range.

#### System unit

**Figure 6.**
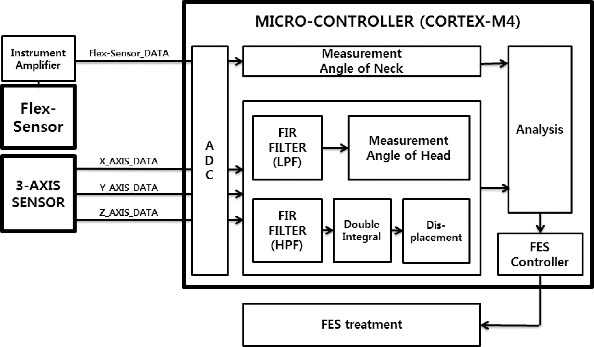
System block diagram.

The whole system has several parts and they are classified by functional structure. The system can be divided into three parts which are mutually associated and each part gives feedback to the others (Figure 6).

#### Instrument

The two sensors complement each other. The three-axis sensor mainly detects the displacement of the head and the flex sensor improves the system's measurement through detecting the angle of the neck. In this unit, many efforts for preventing noise were applied because such electronic circuits are easily affected by many variances.

#### Control

In order to display the vector in a three-dimensional space and diagnose FHP accurately, the system needs a microprocessor with essential functions such as digital signal process (DSP), analogue to digital conversion, etc. The microprocessor Cortex M-4 is specialized in fast DSP and thus it was chosen for the system.

Even if many noise filters are used in analogue electronic circuits, there is still noise signal that is far from the informative signal. The microprocessor significantly reduces the noise in only a few steps and controls all connected devices as received data leads. Due to the fact that the main controller automatically and continuously decides how to deal with treatment, the system unit can be constructed as a portable device.[11,17]

#### Rehabilitation

The FES module will work on adjusting and correcting various faulty postures. Its intensity, duration and application sites will be changed automatically. Because the FES module focuses on moving muscles, more studies on physiotherapy are required to resolve problems effectively.

## Results and discussion

The three-axis acceleration sensor provides constantly varying voltage as the module moves. In fact, there was unnecessary information, acceleration of gravity, and consequently the signal was floating. To compensate the side effect of acceleration of gravity, both analogue and digital filters are used.

**Figure 7.**
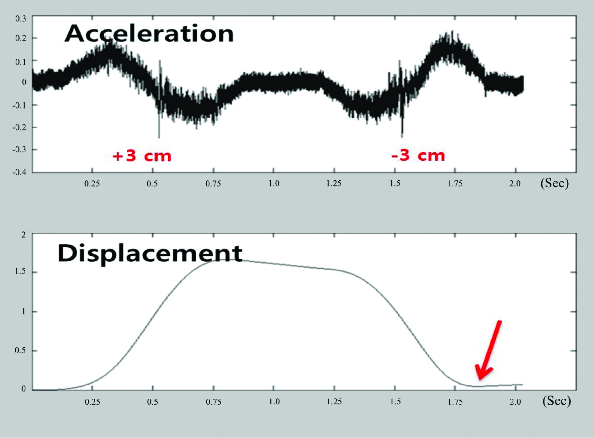
Changes in the displacement of a returning object.

Figure 7 was obtained to confirm whether the calculated displacement is similar with the real distance that the module moved. For these calculations, MATLAB was exploited. The figure presents, respectively, the original acceleration signal on the top and the calculated displacement on the bottom. The original acceleration signal has upward and downward curves, which reflect changes in acceleration due to movement. The phase data give information about the direction of movement: if a rising edge appears first, the direction of movement is positive, while backward motion shows a falling edge first. In its final analysis, this test demonstrates a connection between the movements and displacement.

**Figure 8.**
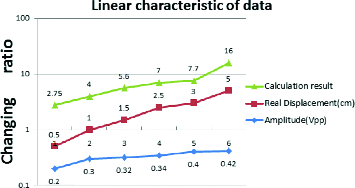
Relative linearity data.

Figure 7 also confirms the accuracy of measurement. If the sensor moves forward and returns to its original position, i.e. the sensor moves 3 cm forward and then also 3 cm backward, the displacement of movement should be zero. To confirm this fact, the measured displacement was compared with the real one to determine whether they are equal. The results shown in the bottom graph in Figure 7 demonstrate that the displacement was concluded to be approximately zero. These experimental results indicate that the measured data are accurate to a considerable degree.

According to Table 3, the calculated results seem not to follow a linear dependence as the real displacement changes. If the data in Table 3 are converted into a logarithmic chart, however, the graph shows proportional characteristics. This is because the differences between the data points increase in a certain proportion. These results indicate that changes in the displacement are connected to alteration in the amplitude of the signal. Moreover, the result after double integration is very similar in shape to the displacement when the result of the double integral is displayed on a logarithmic scale. In other words, the sensor works as expected and it has relatively high precision.

**Table 3.** Calculated data from each subject moved at a different distance.

Subject	1	2	3	4	5	6
Real displacement (cm)	0.5	1	1.5	2.5	3	5
Calculation result (×10^6^)	2.75	4	5.6	7	7.7	16
Amplitude (Vpp)	0.2	0.3	0.32	0.34	0.4	0.42

*The calculation result is a numerical value which is acquired from integral calculation.

The displacement was successfully measured and it was similar to the real one but there were still some unpredicted data. Because the experiment was tested manually, i.e. it was not controlled strictly, the calculated displacement can be slightly different from the displacement that the sensor actually made. A tiny difference may cause a deviation of the result from linearity. In this experiment, however, the deviation is negligible. Therefore, we assume that the calculated displacement is equal to the real displacement.

Thus, the main contribution of the study lies in setting a vector that is composed of many different angles and can be drawn mathematically in three-dimensional space. With the aid of the calculated vector patients would be able to diagnose FHP themselves. Additionally, the device provides guidance for proper adjustment of incorrect posture but future research on setting the vector is required for more precise and effective diagnosis of FHP.

The study, however, has some limitations. The concept of utilizing the vector in analysing and diagnosis of the posture was not studied and there are few published reports. Therefore, it is essential to look for either new investigations on the correlation between the CV angle and the vector angle model or a reliable conversion to the CV angle from the neck angle. Although the simulated data, mainly acquired after double integration, were not accurate enough due to external noise, it was shown that there are several ways to reduce the noise, which would include of course much trial and error and the help of medical experts.

Unlike other devices, this new system will be able to adjust faulty posture immediately with FES. Nevertheless, clinically reliable results of correcting the posture with FES could not be obtained. Further studies on finding the most effective muscle bundles for electrical stimulation are currently under way.

## Conclusions

This study proposes a vector-based approach for diagnosis of FHP by the patients themselves. Additionally, the proposed device would be able to provide proper adjustment for incorrect posture. The vector is the sum of many other vectors and can be drawn mathematically in the three-dimensional space. However, more exhaustive tests need to be carried out with the help of medical experts to calibrate the device to obtain the proportion of angle data from each sensor. This would develop the accuracy and diagnosis range in details.
